# Expression of Toll-like Receptor Genes and Antiviral Cytokines in Macrophage-like Cells in Response to Indole-3-carboxylic Acid Derivative

**DOI:** 10.3390/v16111718

**Published:** 2024-10-31

**Authors:** Alexander Narovlyansky, Alexander Pronin, Vladislav Poloskov, Alexander Sanin, Marina Mezentseva, Irina Fedyakina, Irina Suetina, Igor Zubashev, Felix Ershov, Marina Filimonova, Valentina Surinova, Irina Volkova, Egor Bogdanov

**Affiliations:** 1 National Research Centre for Epidemiology and Microbiology Named after the Honorary Academician N.F. Gamaleya, Ministry of Health of the Russian Federation, Moscow 123098, Russia; en-vladislav@yandex.ru (V.P.); saninalex@inbox.ru (A.S.); marmez@mail.ru (M.M.); irfed2@mail.ru (I.F.); ikas@inbox.ru (I.S.); izubaschev@yandex.ru (I.Z.); felixershov@gmail.com (F.E.); 2 A. Tsyb Medical Radiological Research Center—Branch of the National Medical Research Radiological Center of the Ministry of Health of the Russian Federation, Obninsk 249036, Russia; vladimirovna.fil@gmail.com (M.F.); val_suriniva@mail.ru (V.S.); ik_volkova@mail.ru (I.V.); 3 Faculty of Biotechnology, Lomonosov Moscow University of Fine Chemical Technology, Moscow 119571, Russia; egor.egor.zayats@yandex.ru

**Keywords:** indole-3-carboxylic acid derivative, macrophage-like cells, real-time polymerase chain reaction, gene expression, toll-like receptors, cytokines

## Abstract

Ongoing outbreaks and often rapid spread of infections caused by coronaviruses, influenza, Nipah, Dengue, Marburg, monkeypox, and other viruses are a concern for health authorities in most countries. Therefore, the search for and study of new antiviral compounds are in great demand today. Since almost all viruses with pandemic potential have immunotoxic properties of various origins, particular attention is paid to the search and development of immunomodulatory drugs. We have synthesised a new compound related to indole-3-carboxylic acid derivatives (hereinafter referred to as the XXV) that has antiviral and interferon-inducing activity. The purpose of this work is to study the effect of the XXV on the stimulation of the expression of toll-like receptor genes, interferons, and immunoregulatory cytokines in a macrophage-like cell model. In this study, real-time PCR methods were used to obtain data on the transcriptional activity of genes in macrophage-like cells. Stimulation of the genes of toll-like receptors *TLR2*, *TLR3*, *TLR4*, *TLR7*, *TLR8*, and *TLR9* was detected. A high-fold increase in stimulation (from 6.5 to 16,000) of the expression of the *TLR3* and *TLR4* genes was detected after 4 h of exposure to the XXV. Increased activity of interferon (*IFNA1*, *IFNA2*, *IFNB1*, *IFNK*, and *IFNλ1*) genes with simultaneous stimulation of the expression of interferon receptor (*IFNAR1* and *IFNAR2*) genes and signalling molecule (*JAK1* and *ISG15*) genes was detected. Increased fold stimulation of the expression of the cytokine genes *IL6*, *TNFA*, *IL12A*, and *IL12B* was also observed. Thus, it is shown that the XXV is an activator of *TLR* genes of innate immunity, which trigger signalling mechanisms of pathogen “recognition” and lead to stimulation of the expression of genes of proinflammatory cytokines and interferons.

## 1. Introduction

Ongoing outbreaks and the frequently rapid spread of infections caused by coronaviruses (SARS-CoV-2 and MERS), influenza viruses (H1N1, H1N2, and H5N1), respiratory syncytial virus, Nipah, Dengue, Marburg viruses, echovirus (type 11), monkeypox, and other viruses [1,2] are a concern for health authorities in most countries. Therefore, the search for and study of new antiviral compounds are in great demand today. At the same time, particular attention is paid to the search for and development of immunomodulatory drugs, since almost all of the above viruses have immunotoxic properties of various origins [3].

It is known that the toll-like receptor (TLR) family is involved in the recognition of viruses and initiates innate immunity mechanisms by triggering downstream signalling pathways, inducing the synthesis of interferons (IFNs), other antiviral proteins, and inflammatory cytokines [4], which ultimately leads to the initiation of an adaptive immune response [5]. At the same time, IFNs participate in the regulation of the expression of TLR genes and IFN-stimulated genes (ISGs) [6].

Ten functional TLRs (*TLR1*–10) found in humans are expressed by a variety of immune and non-immune cell types, including fibroblasts, epithelial cells, macrophages, lymphocytes, granulocytes, and dendritic cells [7]. *TLR1*, *TLR2*, *TLR4*, *TLR5*, *TLR6*, and *TLR10* are mainly found on the cell surface and are responsible for identifying microbial lipids, lipoproteins, and proteins. *TLR2* cooperates with *TLR1*, *TLR4*, *TLR6*, and *TLR10* and forms homodimers or heterodimers to recognise different ligands [8]. *TLR4* recognises cell surface lipopolysaccharides (LPSs), myeloid differentiation factor 2 (MD2), and a number of pathogen components that activate the production of inflammatory cytokines [9]. *TLR3*, *TLR7*, *TLR8*, and *TLR9* are localised inside cells (e.g., macrophages and dendritic cells) in compartments such as lysosomes, endosomes, and the intracellular reticulum and can recognise nucleic acids produced by bacteria and viruses, as well as self-nucleic acids produced during cell destruction in autoimmune diseases or cancer [10]. *TLR3* activation occurs in response to the recognition of RNA from destroyed cells, viral double-stranded RNA (dsRNA), and small interfering RNA, which, through the expression of NF-kB, leads to the induction of the synthesis of IFNs and proinflammatory cytokines [11]. *TLR7* recognises viral single-stranded (ss) RNA and induces the production of type I IFNs and proinflammatory cytokines [12,13]. *TLR8* responds to viral and bacterial RNA and triggers the synthesis of IFNs and inflammatory cytokines [14]. *TLR9* identifies bacterial and viral DNA and switches on the synthesis of IFN-α [15].

Among the studied compounds, we can highlight a group of synthesised aminoalkyl esters of 5-methoxyindole-3-carboxylic acid [16]. In 2022–2023, we isolated a compound (6-bromo-1-methyl-5-methoxy-2-(1-piperidinomethyl)-3-(2-diethylaminoethoxy)carbonylindole dihydrochloride, hereinafter referred to as the “XXV”) with antiviral activity against the SARS-CoV-2 virus from this rather extensive group [17]. Since the interferon-inducing activity of this compound was demonstrated and suppression of syncytium formation induced by the spike protein (S-glycoprotein) of SARS-CoV-2 was observed [18], studies continued to investigate its action mechanisms.

The study of the molecular mechanisms of action of promising drug candidates on the innate immune system is necessary for their effective use in medicine to prevent and treat viral, autoimmune, and cancer diseases. Previously, a group of recombinant interferon (IFN) inducers and immunomodulators was studied on a sensitive cellular model of THP-1 monocytes, which turned out to be stimulators of certain genes of toll-like receptors (TLRs) and proinflammatory cytokines [19,20,21,22].

In this study, the effect of the XXV on the stimulation of the expression of genes of TLRs, interferons, some signalling molecules, and cytokines in macrophage-like cells was investigated for the first time.

The purpose of this work is to study the effect of the XXV on the stimulation of the expression of toll-like receptor genes, interferons, and immunoregulatory cytokines in a macrophage-like cell model.

## 2. Materials and Methods

The **THP-1 cell line** [23] (acute monocytic leukaemia, ATCC Catalogue No. TIB 202) was obtained from the Federal State Budgetary Institution “N.N. Blokhin National Medical Research Center of Oncology” of the Russian Ministry of Health. Cells were cultured in RPMI 1640 medium with 10% FBS, glutamine, and antibiotics.

**THP-1 macrophages (THP-PMA-MPH)** were obtained using phorbol 12-myristate 13-acetate (PMA; Cat. No. S-79346-0,005, Sigma-Aldrich, Jerusalem, Israel, prepared in accordance with the manufacturer’s instructions in 98% dimethyl sulfoxide (DMSO)), 50 ng/mL, according to the InvivoGen protocol [24]. Briefly, THP-1 cell suspension was inoculated in 96-well (2 × 10^5^ cells/well) or 6-well (3.2 × 10^6^ cells/well) plates, and PMA was added to each well at a final concentration of 50 ng/mL, incubated for 3 h at 37 °C in 5% CO_2_, and then, after cell attachment, the medium was removed and RPMI 1640 with 10% FBS, glutamine, and antibiotics was added. On day 4, the cells were washed with PBS solution, and nutrient medium was added. For the experiments, 96-well plates were used to determine cytotoxicity, and 6-well plates were used for gene expression.

**The XXV** (6-bromo-5-methoxy-1-methyl-2-(1-piperidinomethyl)-3-(2-diethylaminoethoxy)carbonylindole dihydrochloride) was synthesised at A. Tsyb MRRC using a multi-step scheme [17,18].

The obtained compound was dissolved in RPMI-1640 culture medium and studied in cell cultures at non-toxic concentrations of 12.5 (21.6 µM) and 6.25 μg/mL (10.8 µM).

**A quantitative assessment of the cytotoxicity of the preparations was performed using** MTT (3-[4,5-dimethylthiazol-2-yl]-2,5-diphenyltetrazolium bromide) [25,26]. The culture medium was replaced by RPMI with 2% FBS (Gibco, South America); the XXV was added at an initial concentration of 5 mg/mL (8.6 mM) aqueous solution and titrated in a ratio of 1:2 in a 96-well plate with cells. The titration of the compound was carried out in four replicates to a final dilution of 1:1024. The plate with cells was incubated at 37 °C in 5% CO_2_ for 48 h. After removing the supernatant, 100 μL of 0.5% MTT dye solution (Sigma-Aldrich, USA) was added to each well. Then, incubation was carried out under the same conditions for 2 h (5% CO_2_, 37 °C). The supernatant was carefully removed from the wells, and 100 µL of DMSO was added to dissolve the formed formazan crystals. Cell viability was assessed by the colour intensity of the solution, i.e., by measuring the optical density (OD) at a wavelength of 545 nm using an Immunochem 2100 photometer (High Technology, Inc., North Attleborough, MA, USA). The viability of THP-PMA-MPH cells in the presence of the test compound for 48 h was calculated using GraphPad Prism 6.01. The 50% cytotoxic concentration (CC_50_) values were calculated by generally accepted methods for biological research using the Microsoft Excel 5.0 and GraphPad Prism 6.01 software packages. The working model for CC_50_ analysis was a 4-parameter logistic curve equation (“Nonlinear regression”—“Sigmoidal dose–response (variable slope)” menu items).

### 2.1. Experimental Design

The XXV was dissolved in distilled water at 5 mg/mL (8.6 mM); 10-fold solutions were prepared in RPMI nutrient medium and added to experimental wells with prepared THP-PMA-MPH at final concentrations of 12.5 μg/mL (21.6 μM) and 6.25 μg/mL (10.8 μM). The exposure time of control (free of the XXV) and experimental samples was 4 and 24 h at 37 °C and 5% CO_2_. After incubation, the cells were lysed using the lysis buffer from the RNA isolation kit.

### 2.2. RNA Isolation

Total RNA was isolated using a HiPure Total RNA Plus Kit, Magen Cat. No. R411102 (China), according to the package leaflet of the kit.

### 2.3. Reverse Transcription (Rt) Reaction

The Rt reaction was carried out with the universal primer random 6 on the matrices of total cellular RNA according to the package leaflets of the Reverse Transcription (Rt) Reagent Kit, Cat. No. OT-01, Syntol (Russia). The obtained cDNAs were stored at –70 °C.

### 2.4. Real-Time PCR

The reaction was carried out on a CFX Opus 96 amplifier with a ready-made 2-fold mixture of SSoAdvanced Universal SYBR Green Supermix Cat. No. 1725271 (Bio-Rad, USA) in 0.2 mL microtubes with optically permeable caps (SSIbio, USA). A total of 2 µL of specific (forward and reverse) primer pairs was mixed in a PCR box with 3 µL of cDNA (diluted 2–100 times) and 5 µL of 2x SSoAdvanced Universal SYBR Green Supermix. Each sample was tested in duplicate. PCR programme: 50 °C for 2 min (1 cycle), 95 °C for 10 min (1 cycle), 95 °C for 15 s (1 cycle), then 45 cycles at 95 °C for 15 s, 60 °C for 30 s, and 72 °C for 30 s. Melting programme at the end point of 65–95 °C, step 0.5° C for 10 s. The fluorescence levels of the SYBR Green dye intercalating into DNA were shown on the computer screen (in real time) in the form of DNA amplicon accumulation curves. The quantity was estimated from the threshold cycles (Cq) at the beginning of the logarithmic phase of synthesis. The negative control (cDNA-free sample) did not yield specific PCR products.

**Oligonucleotide PCR primers.** The structures of oligonucleotide primers used in this study were taken from PrimerBank and the published literature. Their structures are shown in Table 1. The oligonucleotides were synthesised by Evrogen (Moscow, Russia).

### 2.5. Gene Expression Level Calculations

The amplification data were processed automatically using CFX Maestro 2.3 (Bio-rad, Hercules, CA, USA). Standard deviations (±SDs) of Cq values of the logarithmic phase and the change in levels in the test samples (delta Cq ± SDs) were determined. The 18S ribosomal RNA gene was used as a stable reference normaliser of gene expression. Specificity of DNA products was determined by T melting. Changes in gene activity (2deltaCq) in experimental cell samples were determined relative to control ones, which were taken to be equal to 1.

## 3. Results

### 3.1. Determination of Cytotoxicity of the XXV Compound

**Figure 1:**
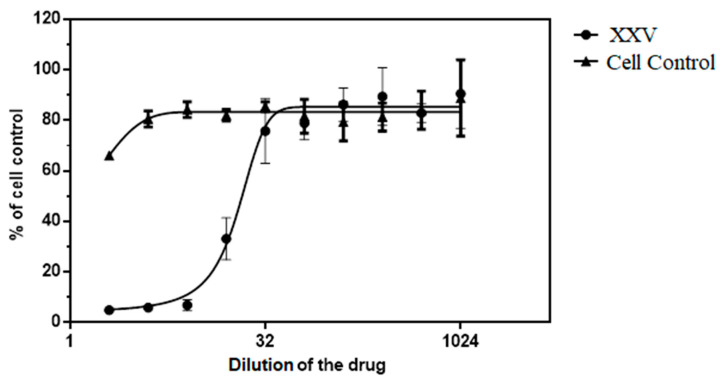
Determination of the cytotoxic effect of the XXV 48 h after addition to the THP-PMA-MPH cell culture (using the vital MTT dye). CC_50_ = 256.94 μg/mL (445.3 μM). The optical density (OD) was assessed at a wavelength of 545 nm using an Immunochem 2100 photometer (USA). The 50% cytotoxic concentration (CC_50_) was calculated using GraphPad Prism 6.01. The abscissa axis showed the dilution of the XXV (initial concentration of 5 mg/mL (8.6 mM), and the ordinate axis showed the percentage (%) of living cells.

Based on the data obtained in the study of the cytotoxic effect of the XXV on THP-PMA-MPH cells using the vital MTT dye, an analytical curve was constructed from which the CC_50_ value was determined. The concentration that reduced the optical density by 50% compared to the control was 256.94 μg/mL (445.3 μM) (Figure 1).

Further, all studies were performed at XXV concentrations of 6.25 and 12.5 μg/mL, which did not have a cytotoxic effect on the THP-PMA-MPH cells.

### 3.2. Effect of the XXV on TLR Gene Expression

Human cell TLRs are expressed at the plasma membrane (e.g., *TLR1*, *TLR5*, *TLR6*, and *TLR10*), in intracellular endosomes (e.g., *TLR3* and *TLR7*–9), or in both compartments (e.g., *TLR2* and *TLR4*) [5,8], and their localisation critically regulates TLR signalling [8]. This study is focused on TLRs, where the expression of genes is associated with the activation of antiviral immunity and the induction of IFNs and inflammatory cytokines. In a preliminary series of experiments, we analysed the expression level of the RIG-1 and MDA5 genes at XXV concentrations of 25 μg/mL, 12.5 μg/mL, 6.25 μg/mL, 3.15 μg/mL, and 1.56 μg/mL. The maximum expression level was observed at concentrations of 12.5 μg/mL and 6.25 μg/mL. This was the reason why the XXV was tested at the above concentrations of 12.5 μg/mL and 6.25 μg/mL.

The effect of the XXV compound on the expression levels of innate immune TLR genes in THP-PMA-MPH is presented in Table 2.

**Figure 2:**
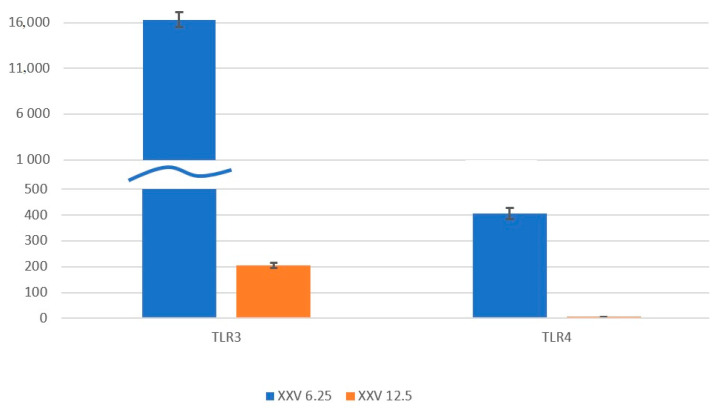
Relative transcription level of the *TLR3* and *TLR4* genes 4 h after stimulation with the compound XXV in THP-PMA-MPH. The data obtained from the analysis in two replicates are presented. The calculations were performed relative to the control cells not treated with the XXV with normalisation to the reference gene *ribRNA*. The amplification data were processed automatically using CFX Maestro (Bio-rad, Hercules, CA, USA). The standard deviations (±SDs) of the Cq values of the logarithmic phase and the change in levels in the test samples (delta Cq ± SDs) were determined. The changes in gene activity (2deltaCq) in the experimental cell samples were determined relative to the control ones, which were taken to be equal to 1. The abscissa axis shows the expressed genes when treated with the XXV at concentrations of 6.25 μg/mL (10.8 µM) and 12.5 μg/mL (21.6 µM); the ordinate axis shows the relative expression level.

In differentiated THP-PMA-MPH, the XXV at concentrations of 6.25 and 12.5 μg/mL activated a number of *TLR* genes associated with the functioning of the IFN system, cytokines, and innate immunity. For example, stimulation of the *TLR2*, *TLR3*, *TLR4*, *TLR7*, *TLR8*, and *TLR9* genes was detected. At the same time, attention is drawn to the fold increase in stimulation of the *TLR3* gene (increase in stimulation of more than 16,000 times) and the *TLR4* gene (increase in stimulation of more than 400 times) detected at 4 h of exposure to the XXV. We believe that we observe cooperation of the inclusion of gene expression of endosomal TLRs with, apparently, the leading role of *TLR3*. This study shows that intense stimulation of *TLR* genes occurs mainly at early stages of exposure (4 h) to the XXV (Figure 2), and after 24 h of treatment, we no longer observe stimulation of the expression of most *TLR* genes. It should be noted that the level of stimulation of *TLR* gene expression also depended on the concentration of the XXV and was more pronounced at a concentration of 6.25 μg/mL.

### 3.3. Effect of the XXV Compound on the Interferon Gene Expression

Stimulation of the expression of a number of type I *IFN* genes, namely *IFNA1*, *IFNA2*, *IFNB1*, *IFNK*, and *IFNW1*, by 2–12 times was observed (Table 3). In addition, stimulation of the expression of type III *IFN* genes was detected. The fold increase in the stimulation of the *IFNλ1* and *IFNλ3* genes was more than two times (Table 3). Here, the fold increase in the stimulation of the gene expression was less expressed compared to the stimulation of the *TLR* genes and was observed at different periods of exposure to the XXV. We do not consider the 1.87-fold stimulation of the *IFNG* gene detected 4 h after treating the cells with the XXV to be reliable, especially since cells of macrophage origin usually do not produce IFN-γ.

### 3.4. Effect of the XXV on the Expression of Immunoregulatory Cytokine Genes

When treating THP-PMA-MPH cells with the XXV, stimulation of the expression of genes of *TNFA* (increase in stimulation of more than 4 times) and *IL6* proinflammatory cytokines (increase in stimulation of more than 200 times) was detected. We noted stimulation (3-300-fold, depending on the exposure time and concentration of the XXV) of the expression of *IL12*–*IL12A* and *IL12B* immunoregulatory cytokine genes (Table 4).

### 3.5. Effect of the XXV on the Expression of Interferon Receptor Genes and Signalling Molecules Involved in Immune Signal Transmission

When treating THP-PMA-MPH cells with the XXV, stimulation of the expression of interferon alpha receptor (*IFNAR1* and *IFNAR2*) genes was detected after 4 h of exposure at both concentrations tested. Stimulation (more than 10-fold) of the expression of the *JAK1* gene, which plays a critical role in initiating responses for several major cytokine receptor families, was also detected. After 4 h, but not after 24 h, of exposure to the XXV, stimulation (maximum 14.39-fold at an XXV concentration of 6.25 μg/mL) of the *ISG15* gene expression was detected. ISG-15 is induced by type I IFNs and has multiple functions. The exact functions are diverse and vary across species, but include potentiation of interferon gamma (type II IFN) production in lymphocytes, ubiquitin-like conjugation of newly synthesised proteins, and negative regulation of the type I IFN response. It exhibits antiviral activity against both DNA and RNA viruses, including influenza A, HIV-1, and Ebola viruses [28,29].

In addition, we studied the expression of the *IRAK-3* (interleukin-1 receptor-associated kinase 3) gene, which encodes a member of the interleukin-1 receptor-associated protein kinase family. Members of this family are important components of the Toll/IL-R immune signalling pathways. This protein is mainly expressed in monocytes and macrophages and functions as a negative regulator of toll-like receptor signalling [30]. A high-fold increase in stimulation of this gene (by 47 times) was detected (Table 5).

## 4. Discussion

The studied TLR genes encode receptors that recognise structural components of RNA- and DNA-containing viruses, many of which cause dangerous diseases. The susceptibility, nature of development, and outcome of an infectious process largely depend on the expression levels of innate immunity receptors [4]. At the same time, the processes of induction of *TLR* and *IFN* genes, and then the action of IFNs, are interconnected, since the expression levels of *TLR* genes are regulated by synthesised IFNs [6]. The study of the mechanisms of induction of type I IFN is usually carried out using viruses (*Sendai virus*, *Newcastle disease virus*, *Semliki Forest virus*, etc.) and various IFN inducers (Poly (I:C), Poly (AÛ), dsRNA, etc.), while mitogens (PHA, conA, LPS, and PWM) necessary for the study of IFN-γ are not used. In our study on non-polarised macrophages, we investigated the effect of the XXV at non-toxic concentrations, at which immunomodulatory activity expressed by the stimulation of expression of innate immunity genes (*TLR* and *IFN* genes and proinflammatory cytokines) was observed.

After differentiation of PMA, we obtained non-polarised (non-activated) macrophages that were used to study the effect of the XXV on the level of gene expression. Apparently, this may be the reason for a wide range of values of gene expression stimulation levels. The obtained results seem to indicate that the polarisation of macrophages followed the M1 pathway—this fact is supported by the stimulation of the *TNFa*, *IL6*, and *IL12* genes. In our opinion, when continuing this work, in order to differentiate THP-1 cells, it is more optimal to use low doses of PMA (10–20 ng/mL), as suggested by a number of authors [31,32]. The MPHs formed under these conditions are immature or weakly activated and can be used for polarisation in M1 and M2 phenotypes. Then, apparently, high phagocytic activity and expression of surface markers of cellular differentiation of CD14, CD35, *TLR2*, and CD11b/CD18 could show us the differentiation level of THP-MPH. In addition, as a continuation of this work, experiments should be carried out using THP-PMA-MPH virus-infected cells, since it is unknown how the XXV will behave in the presence of viruses and the IFN antagonists they induce. At the same time, it will be possible to study the expression of other *ISGs* (e.g., *IFIT1*, *MX1*, and *OAS1*) and genes encoding signalling molecules responsible for conducting the XXV-induced intracellular signal.

In a preliminary series of experiments (see above), we analysed the expression level of the *RIG-1* and *MDA5* genes in THP-PMA-MPH cells at different XXV concentrations. The maximum expression level (2.75- and 1.84-fold stimulation of expression for *RIG1* and 3.94- and 3.18-fold stimulation of expression for *MDA 5*) was observed 4 h after treatment with the XXV at concentrations of 12.5 μg/mL and 6.25 μg/mL, respectively (unpublished results). These results showed that the XXV induces the expression of not only *TLR* genes but also other receptor genes involved in the antiviral response.

The tables present data on the stimulation of expression of a group of innate immunity genes by the XXV in PMA-stimulated human THP-1 monocytes, obtained as PCR threshold cycle (Cq) values presented as a fold increase in gene stimulation levels. The XXV stimulated the expression of not only *TLR* genes but also *IFNA*, *IFNB*, *IFNK*, and *IFNλ* genes, as well as IFN receptor genes (*IFNAR1* and *IFNAR2*), apparently leading to the activation of downstream IFN signalling pathways through JAK-1 and activating IFN-dependent genes, such as IFN-stimulated gene 15 (*ISG15*). The development of an antiviral state in cells is associated with the expression of *IFN* genes. It should be noted that in this study we detected stimulation of the expression of the *IRAK3* gene, which negatively correlated with the expression of inflammatory cytokines (IL-6 and TNF-α) [33]. This may suggest that the XXV may not only trigger the expression of innate immune genes but also activate immunoregulatory genes that control an excessive immune response. Since it is known that the TLR signalling pathway is tightly controlled and multiple negative regulators of TLR signalling are present at different levels to maintain immune homeostasis, it is necessary to further study the effect of this compound on TLR signalling pathway inhibitors (IRAK-M, SOCS-1, etc.) [5].

It should be noted that the level of stimulation of *TLR* gene expression also depended on the concentration of the XXV and was more pronounced at a concentration of 6.25 μg/mL. We observed this effect not only for *TLR* genes but also for other genes (*IL6*, *IL12A*, *IFNAR1*, *IFNAR2*, *JAK1*, *ISG15*, and *IRAK3*). It might be that a lower concentration of the compound is optimal and higher concentrations are inhibiting, but this suggestion needs additional study.

Previously, constitutive expression of genes of six TLR types was determined in THP-1 monocytes using quantitative real-time PCR: in *TLR2* and *TLR4* (present on the cell surface) and in *TLR3*, *TLR7*, *TLR8*, and *TLR9* (localised in the endosomes). The authors found that the transcription levels of the studied *TLR* genes in THP-1 monocytes differed significantly. The most transcriptionally active genes in THP-1 monocytes were *TLR4* and *TLR8* (Cq22–26), and the least active genes were *TLR2*, *TLR3*, *TLR7*, and *TLR9* (Cq30–40) [19,20,21,22].

The authors showed that THP-1 monocytes exposed to the Cytokine-Stimul-Best reagent differentiate into macrophage-like cells, causing a predominant increase in the transcriptional activity of the *TLR3* and *Stat1b* genes, which are involved in the induction and action of IFNs [34].

In addition, it is known that the interaction of TLR adapters and kinases (IRAK 1–4) leads to the activation of NF-kB and MAP kinases, the main regulators of cytokine and interferon transcription [35,36]. The *IRAK3* gene encodes a member of the interleukin-1 receptor-associated kinase protein family. Members of this family are important components of the Toll/IL-R immune signalling pathways. This protein is predominantly expressed in monocytes and macrophages and acts as a negative toll-like receptor signalling regulator. Therefore, the detection of stimulation of *IRAK3* gene expression confirms the involvement of the XXV in the stimulation of *TLR* gene expression with subsequent activation of negative toll-like receptor signalling regulation. The detection of increased activity of *IFN* (*IFNA1*, *IFNB1*, *IFNK*, and *IFNλ1*) genes with simultaneous stimulation of genes of IFN (*IFNAR1* and *IFNAR2*) receptors and some genes of signalling molecules that determine its action (*JAK1* and *ISG15*), as well as a fold increase in the stimulation of the *TNFA*, *IL6*, *IL-12A*, and *IL-12B* cytokine genes, apparently, may indicate the ability of the XXV to stimulate expression of innate immunity genes in macrophage cells, which are necessary for triggering processes that lead to the successful elimination of foreign genetic information and counteraction to pathogenic microorganisms.

Thus, macrophage-like cells responded to treatment with the XXV by increasing the expression of the *TLR2*, *TLR3*, *TLR4*, *TLR7*, *TLR8*, and *TLR9* genes. This is consistent with the pronounced inflammatory response of activated macrophages. The *TLR2*, *TLR3*, *TLR4*, *TLR7*, *TLR8*, and *TLR9* receptors have different specific ligands, cellular localisation, associated adaptor proteins, and signalling pathways. The XXV, being essentially an IFN inducer, stimulated IFN-alpha, IFN-beta, and IFN-dependent genes, which ultimately led to the development of an antiviral state in the cells.

It should be noted that in this study we did not aim to show whether the XXV is an agonist of certain types of TLRs. Such conclusions require separate special studies.

The list of known TLR agonists continues to expand with new natural and synthetic compounds [37]. The prospect of using TLR agonists in combination with immune checkpoint inhibitors and antitumour vaccines provides new opportunities for the treatment of malignant tumours [38]. Moreover, compounds differing from canonical TLR agonists in chemical structure but also capable of direct interaction with TLRs have already been discovered among a large group of immunomodulators [39].

The XXV is an activator of *TLR* genes of innate immunity, which trigger signalling mechanisms of pathogen “recognition” and lead to the synthesis of proinflammatory cytokines and IFNs. The ability to activate the expression of *TLR* genes is an undoubted advantage of the drug. It expands the range of the drug’s use in viral and bacterial infections, and possibly in autoimmune and cancer diseases. Macrophages play a critical role in early defence responses to viral pathogens and influence dendritic cells as well as T- and B-lymphocyte functions. Stimulation of *TLR* gene expression in macrophages by the XXV increases their sensitivity to pathogens of various natures and, apparently, causes a stronger immune response in the body.

## 5. Conclusions

Stimulation of the genes of the toll-like receptors *TLR2*, *TLR3*, *TLR4*, *TLR7*, *TLR8*, and *TLR9* was detected. A high-fold increase in stimulation (from 6.5 to 16,000) of the expression of the *TLR3* and *TLR4* genes was detected after 4 h of exposure to the XXV. Increased activity of *IFN* (*IFNA1*, *IFNA2*, *IFNB1*, *IFNK*, and *IFNλ1*) genes with simultaneous stimulation of the expression of IFN receptor (*IFNAR1* and *IFNAR2*) genes and genes of signalling molecules (*JAK1* and *ISG15*) that determine the action of IFNs was detected. The fold increase in stimulation of the cytokine genes *TNFA*, *IL6*, *IL12A*, and *IL12B* was also observed.

Thus, it has been shown that the XXV is an activator of *TLR* genes of innate immunity, triggering signalling mechanisms of pathogen “recognition” and leading to the synthesis of proinflammatory cytokines and IFNs. The ability to activate the expression of *TLR* genes is an undoubted advantage of the drug. It expands the range of the drug’s use in viral and bacterial infections. Macrophages play a critical role in early defence responses to viral pathogens and influence dendritic cells as well as T- and B-lymphocyte functions. Stimulation of *TLR* gene expression in macrophages by the XXV increases their sensitivity to pathogens of various natures and causes a stronger immune response in the body.

## Figures and Tables

**Table 1:** Structure of oligonucleotide primers.

Gene Name	Sequence	Source
*TLR2*	F:ATCCTCCAATCAGGCTTCTCT R:GGACAGGTCAAGGCTTTTTACA	NM_003264.5
*TLR3*	F:TTGCCTTGTATCTACTTTTGGGG R:TCAACACTGTTATGTTTGTGGGT	NM_003265.3
*TLR4*	F:AGACCTGTCCCTGAACCCTAT R:CGATGGACTTCTAAACCAGCCA	NM_138557.3
*TLR7*	F: TCCTTGGGGCTAGATGGTTTC R: TCCACGATCACATGGTTCTTTG	NM_016562.4
*TLR8*	F: ATGTTCCTTCAGTCGTCAATGC R: TTGCTGCACTCTGCAATAACT	NM_138636.5
*TLR9*	F: AATCCCTCATATCCCTGTCCC R: GTTGCCGTCCATGAATAGGAAG	NM_017442.4
*IFNA1*	F: GCCTCGCCCTTTGCTTTACT R: CTGTGGGTCTCAGGGAGATCA	NM_024013.3
*IFNA2*	F: GCTTGGGATGAGACCCTCCTA R: CCCACCCCCTGTATCACAC	NM_000605.4
*IFNB1*	F:GCTTGGATTCCTACAAAGAAGCA R: ATAGATGGTCAATGCGGCGTC	NM_002176.4
*IFNE*	F:GGCCTCTACCACTATCTTCTCTC R:ACACTGCTGAATTGACAAGGTTT	NM_176891.5
*IFNK*	F: GTGGCTTGAGATCCTTATGGGT R: CAGATTTTGCCAGGTGACTCTT	NM_020124.3
*IFNW1*	F:GAAGGCCCATGTCATGTCTGT R:GAGTTGGTCTAGGAGGGTCAT	NM_002177.3
*IFNG*	F: TCGGTAACTGACTTGAATGTCCA R: TCGCTTCCCTGTTTTAGCTGC	NM_000619.3
*IFNλ1*(*IL29*)	F:CACATTGGCAGGTTCAAATCTCT R:CCAGCGGACTCCTTTTTGG	NM_172140.2
*IFNλ3*(*IL29B*)	F: TAAGAGGGCCAAAGATGCCTT R: CTGGTCCAAGACATCCCCC	NM_172139.4
*TNF-α*	F:ATGATGGCTTATTACAGTGGCAA R:GTCGGAGATTCGTAGCTGGA	NM_000576.3
*IL-6*	F:ACTCACCTCTTCAGAACGAATTG R:CCATCTTTGGAAGGTTCAGGTTG	NM_000600.5
*IL12A*	F: CCTTGCACTTCTGAAGAGATTGA R: ACAGGGCCATCATAAAAGAGGT	NM_000882.4
*IL12B*	F: ACCCTGACCATCCAAGTCAAA R:TTGGCCTCGCATCTTAGAAAG	NM_002187.3
*IFNAR1*	F: ATTTACACCATTTCGCAAAGCTC R: TCCAAAGCCCACATAACACTATC	NM_000629.3
*IFNAR2*	F: TCATGGTGTATATCAGCCTCGT R: AGTTGGTACAATGGAGTGGTTTT	NM_207585.3
*Jak1*	F: CCACTACCGGATGAGGTTCTA R: GGGTCTCGAATAGGAGCCAG	NM_002227.4
*ISG15*	F: GCGCAGATCACCCAGAAGAT R: GTTCGTCGCATTTGTCCACC	[27]
*IRAK3*	F:TGCGGGATCTCCTTAGAGAA R:GCAGAGAAATTCCGAGGGCA	NM_007199.3
*18S PHK*	F GTAACCCGTTGAACCCCATT R CCATCCAATCGGTAGTAGGCG	NR_003286

**Table 2:** Expression of *TLR* genes in THP-PMA-MPH cells treated with the XXV compound.

Gene Name	Fold Increase in the Stimulation of Gene Expression Levels			
**Drug Concentration**	**6.25 μg/mL (10.8 µM)**		**12.5 μg/mL (21.6 µM)**	
**Exposure Time**	**4 h**	**24 h**	**4 h**	**24 h**
*TLR2*	93.67	0.1	1.42	0.6
*TLR3*	16,312.25	N/A	205.5	0.3
*TLR4*	406.02	0.1	6.59	1
*TLR7*	33.39	0.2	0.3	0.6
*TLR8*	78.17	0.2	1.44	0.8
*TLR9*	5.55	0.09	0.2	3.4

Note: The amplification data were processed automatically using CFX Maestro (Bio-rad, Hercules, CA, USA). The standard deviations (±SDs) of the Cq values of the logarithmic phase and the change in levels in the test samples (delta Cq ± SDs) were determined. The changes in gene activity (2deltaCq) in the experimental cell samples were determined relative to the control ones, which were taken to be equal to 1. N/A means no expression detected.

**Table 3:** Expression of *IFN* genes in THP-PMA-MPH cells treated with XXV.

Gene Name	Fold Increase in the Stimulation of Gene Expression Levels			
**Drug Concentration**	**6.25 μg/mL**		**12.5 μg/mL**	
**Exposure Time**	**4 h**	**24 h**	**4 h**	**24 h**
*IFNA1*	2.09	1.24	10.73	0.71
*IFNA2*	1.18	1.06	0.1	8.8
*IFNB1*	3.18	N/A	2.66	2.0
*IFNE*	N/A	N/A	1	N/A
*IFNK*	0.37	2.59	12.03	11.59
*IFNW1*	2.0	N/A	0.16	1.27
*IFNG*	1.87	N/A	N/A	N/A
*IFNλ1*	N/A	N/A	2.7	N/A
*IFNλ3*	0.23	0.57	0.44	2.17

Note: The amplification data were processed automatically using CFX Maestro (Bio-rad, Hercules, CA, USA). The standard deviations (±SDs) of the Cq values of the logarithmic phase and the change in levels in the test samples (delta Cq ± SDs) were determined. The changes in gene activity (2deltaCq) in the experimental cell samples were determined relative to the control ones, which were taken to be equal to 1. N/A means no expression detected.

**Table 4:** Expression of immunoregulatory cytokine genes in THP-PMA-MPH cells after treatment with XXV.

Gene Name	Fold Increase in the Stimulation of Gene Expression Levels			
**Drug Concentration**	**6.25 μg/mL**		**12.5 μg/mL**	
**Exposure Time**	**4 h**	**24 h**	**4 h**	**24 h**
*TNFA*	1.8	N/A	4.4	N/A
*IL6*	213.85	N/A	28.7	N/A
*IL12A*	45.39	0.07	19.8	0.08
*IL12B*	N/A	321.76	N/A	3.37

Note: The amplification data were processed automatically using CFX Maestro (Bio-rad, Hercules, CA, USA). The standard deviations (±SDs) of the Cq values of the logarithmic phase and the change in levels in the test samples (delta Cq ± SDs) were determined. The changes in gene activity (2deltaCq) in the experimental cell samples were determined relative to the control ones, which were taken to be equal to 1. N/A means no expression detected.

**Table 5:** Expression of genes encoding signalling molecules involved in immune signal transduction in THP-PMA-MPH cells treated with XXV.

Gene Name	Fold Increase in the Stimulation of Gene Expression Levels			
**Drug Concentration**	**6.25 μg/mL**		**12.5 μg/mL**	
**Exposure Time**	**4 h**	**24 h**	**4 h**	**24 h**
*IFNAR1*	46.24	0.12	7.53	0.37
*IFNAR2*	7.0	N/A	2.14	N/A
*JAK1*	10.73	0.44	3.93	0.45
*ISG15*	14.39	1.18	3.97	1.46
*IRAK3*	47.93	0.18	13.67	0.47

Note: The amplification data were processed automatically using CFX Maestro (Bio-rad, Hercules, CA, USA). The standard deviations (±SDs) of the Cq values of the logarithmic phase and the change in levels in the test samples (delta Cq ± SDs) were determined. The changes in gene activity (2deltaCq) in the experimental cell samples were determined relative to the control ones, which were taken to be equal to 1. N/A means no expression detected.

## Data Availability

The original contributions presented in this study are included in this article; further enquiries can be directed to the corresponding author(s). The raw data supporting the conclusions of this article will be made available by the authors upon request.
